# Investigation on peculiar SNR variation at TianMa radio telescope

**DOI:** 10.1038/s41598-019-55072-3

**Published:** 2019-12-05

**Authors:** Qingbao He

**Affiliations:** 1 State Key Laboratory of Information Engineering in Surveying, Mapping and remote sensing, Wuhan University, Wuhan, 430070 China; 2 Shanghai Astronomical Observatory, Chinese Academy of Sciences, Shanghai, 200030 China

**Keywords:** Rings and moons, Astronomical instrumentation

## Abstract

Chinese lunar spacecraft Chang’E-3 (CE3) and Chang’E5-T1 (CE5T1) were launched in 2013 and 2014, respectively and very long baseline interferometry (VLBI) observations were performed. Signal to noise ratio (SNR) of the Tianma (TM) station experienced peculiar variation, whereas SNRs of other stations were rather stable. Further, it happened only when observing spacecraft, and showed no such variation when performing astronomical observations. Moreover, it was distinctive on X band signals, whereas it was not noticed on S band signals. Analysis showed that the SNR variation was closely related with changing rate of elevation angle. Further investigations discovered that there were two sets of antenna control software at TM station, and the SNR variation originated from a bug in elevation control software used for observing spacecraft, not for astronomical observations. The bug caused big pointing error (around ± 30 as) on elevation angle of TM, which resulted in the peculiar SNR variation. It was not noticed on S band signals due to its relatively wide and flat main beam comparing with ± 30 as pointing error. However, the bug was fixed in software update in July, 2016, and the SNR of TM showed no such variation in 2017 and 2018.

## Introduction

Tianma (TM) radio telescope is a newly-built fully-steerable instrument located in Shanghai, China^1^. It has a main reflector of 65 m in diameter. The construction was completed in October, 2012. Since then, TM performed comprehensive observations, including astronomical observations of molecular spectral line emission^2^, pulsars^3^, and various radio sources^4^. Apart from these, TM also performed observations on spacecraft for deep space exploration, such as Chang’E-3 (CE3) and Chang’E-5-T1 (CE5T1).

CE3 was the third Chinese lunar spacecraft, which was launched on December 1, 2013, and started orbiting the Moon on December 6, 2014. It made a soft landing on the Moon on December 14, 2013^5^. CE5T1 was an experimental lunar mission to conduct atmospheric re-entry test, and was launched on October 23, 2014^6,7^. It consisted of a return vehicle and a service module. On October 31, 2014, the return vehicle went back to Earth, while the service module continued its extended tests in space. In both missions, very long baseline interferometry (VLBI) observations were performed to improve the accuracy of spacecrafts orbit determination, which involved telescopes of Tianma (TM, 65 m), Beijing (BJ; 50 m), Kunming (KM; 40 m), and Urumuqi (UR; 25 m). On April 29, 2015, VLBI observations were performed both on CE3 and CE5T1, for their angular separation was very small.

In the observations of spacecraft, the signal-to-noise ratio (SNR) of TM experienced peculiar variation all the time, with the variation period changing from a few tens of seconds to a few hundreds of seconds and the variation amplitude reaching a few dB, whereas the SNRs of other stations were rather stable. What’s more, such phenomenon did not appear when TM performed astronomical observations.

TM is very sensitive to changes in radiation power of observation target, and changes in directional pointing of telescope itself, because of its large diameter (65 m). Unstable radiation power from the spacecraft antenna did not seem to be the cause, for other stations did not experience such variation at all. Error in pointing of TM could not properly explain why it occurred only when observing spacecraft, and not when performing astronomical observations. Therefore, it could be caused by a new astronomical event. However, it could also be caused by instrumental or anthropogenic reasons. Whatever the cause is, it is necessary to find out the reason, and thorough Investigations are needed before drawing a conclusion.

Below, in Section 2, we show the peculiar SNR variation of TM when observing spacecraft. In section 3, the relationship between elevation angle and SNR variation is discussed. In section 4, the SNRs of CE3 and CE5T1 on April 29, 2015 are analyzed, and the pointing error of TM is considered as the cause for SNR variation. In Section 5, actual elevation and azimuth data from log files of TM are investigated, and prove the SNR variation was caused by error on actual elevation angle, which originated from a bug in elevation control software. Section 6 concludes this paper. Time in this paper is in coordinated universal time (UTC).

## Peculiar SNR Variation

**Figure 1:**
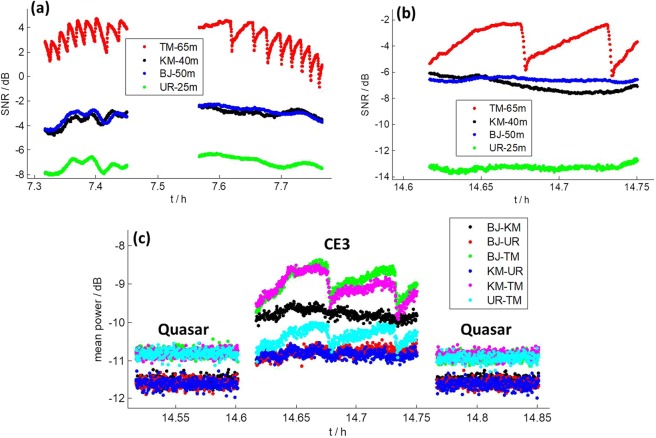
SNR in X band DOR signals of CE3 (**a**) on December 8, 2013 and (**b**) on December 9, 2013. (**c**) Mean value of cross correlation power of quasar and CE3 on December 9, 2013.

In the CE3 mission, before landing on the Moon, CE3 transmitted DOR (Differential One-way Range) signals in X band, and VLBI observations were performed. The SNRs of VLBI stations were calculated, in periods of both before landing on the Moon and after landing on the Moon. The frequency setting and SNR calculation methods can be found in^5^. The interesting phenomenon was that the SNR of TM experienced peculiar variation all the time, which was not found at the other 3 stations. This excludes unstable transmitting power of the spacecraft antenna to be the cause. Figure 1a,b present the SNR results of December 8, 2013, and December 9, 2013, respectively, when CE3 was orbiting the Moon. The SNR results after CE3 landing on the Moon will be presented in section 3. The gap (around 5 minutes) in Fig. 1a was caused by observing quasars for calibration. Indeed, before CE3 landed on the Moon, Chinese VLBI stations observed CE3 and quasars alternately.

It is obvious that the SNR of TM has a distinctive variation in Fig. 1a,b, which did not appear at other stations. Further, the SNR of TM remarkably declines 3.5 dB in amplitude within 15 seconds (Fig. 1b), and the variation period changes with time (Fig. 1a). What’s more, in Fig. 1a, the variation patterns of the two scans of TM show a symmetric behavior: in the first the SNR decreases slowly and increases abruptly, while in the second it increases slowly and decreases abruptly. Such kind of phenomenon occurred in all the observations of all spacecrafts observed in the years of 2013, 2014, and 2015. More SNR results will be presented later in this paper.

At first, antenna’s pointing error because of wind was considered as a possible cause of this variation. In this case, the variation should also occur when performing astronomical observations. However, we did not notice such variation in the past 6 years when observing astronomical targets. Here we present results of observing a quasar during the CE3 mission in Fig. 1c. In the case of observing the quasar, signal power from the quasar is mixed with the antenna noise power, which resulted in total noise power increasing. We didn’t calculate the total noise power, because the AGC (Automatic Gain Control) unit, which kept the total power as a constant, was operated in the receiving system of each station^5^. Instead, we calculated the mean value of cross correlation power between 2 stations, which was also sensitive to changes in the noise power of antenna itself and signal power from observation target. Figure 1c shows results of 6 baselines on December 9, 2013. The middle part is the result of observing CE3. The other two parts are results of observing a quasar (0003–066). As we can see in the middle part, the results of BJ-TM, KM-TM, and UR-TM have the same changing pattern as SNR of TM in Fig. 1b, which indicates they were caused by power change of TM. However, in the first and third parts (observing quasar) of Fig. 1c, results of all baselines are rather stable. This proves the variation only occurred when observing the spacecraft, and not when observing astronomical targets. Therefore, pointing error because of wind is excluded to be the reason.

**Figure 2:**
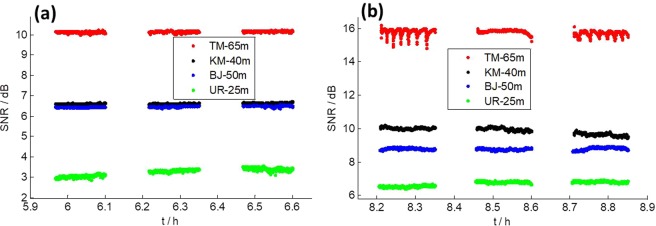
SNR of CE5T1 on October 26, 2014. (**a**) S band. (**b**) X band

In the CE5T1 mission, CE5T1 transmitted both X and S band signals^8^, which were received simultaneously by Chinese VLBI stations, for they were equipped with S/X receivers. Figure 2 shows the SNR of CE5T1 on October 26, 2014, when it was in its Earth-Moon transfer orbit. Figure 2a,b show the SNR of S and X band signals, respectively. It is obvious that the variation happened again on TM station, but strangely only on X band signals, not on S band signals. The SNR variation of TM in Fig. 2b shows the same character as in Fig. 1a.

**Figure 3:**
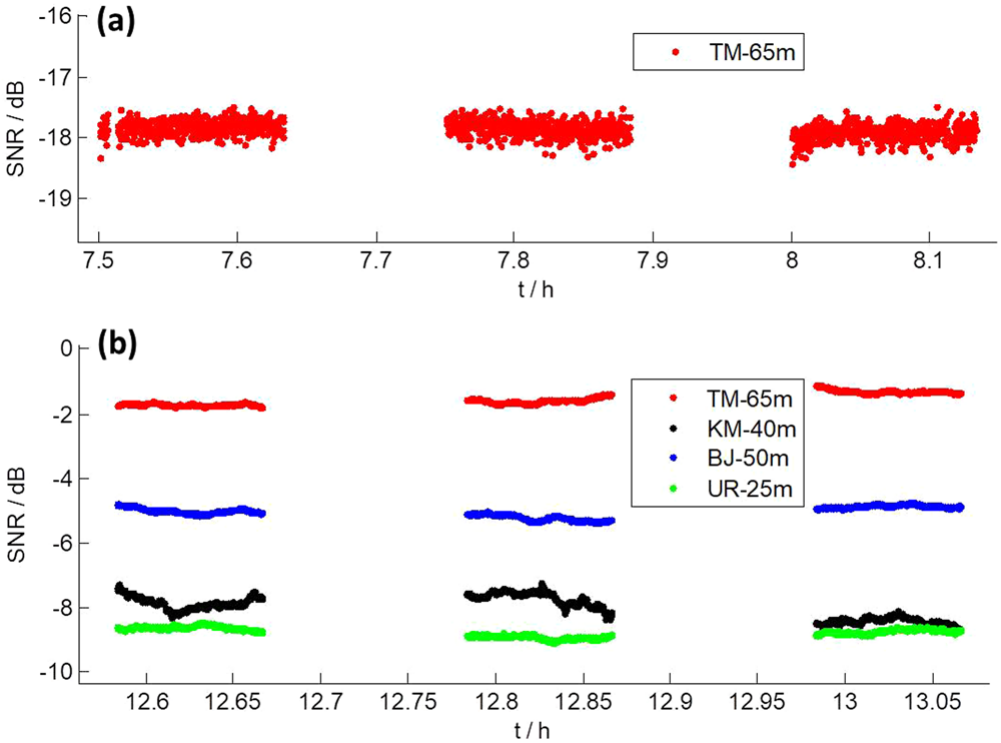
(**a**) SNR in X band signals of MRO on March 29, 2017. (**b**) SNR in X band DOR signals of CE3 on November 19, 2018

In the year 2016, no data were collected for observing spacecraft. However, observations of spacecraft were performed again in 2017 and 2018. On March 29, 2017, TM pointed to Mars and received X band signals from the Martian spacecraft MRO (Mars Reconnaissance Orbiter)^9^, which was launched by NASA. On November 19, 2018, Chinese VLBI stations performed observation on CE3 again, and received X band DOR signals. Their SNR values were calculated and shown in Fig. 3a,b, respectively. Surprisingly, the variation didn’t occur in both of the two observations.

In summary, the peculiar variation occurred in the SNR of TM in the years 2013 and 2014, when TM was observing spacecrafts CE3 and CE5T1. Such variation didn’t occur when TM was observing astronomical targets. The SNR variation was distinctive in X band signals, and was not noticed in S band signals. Further, it disappeared in the years 2017 and 2018. It could not be explained by antenna’s pointing error because of wind or unstable transmitting power of the spacecraft antenna.

## Relation between Elevation and SNR Variation

**Figure 4:**
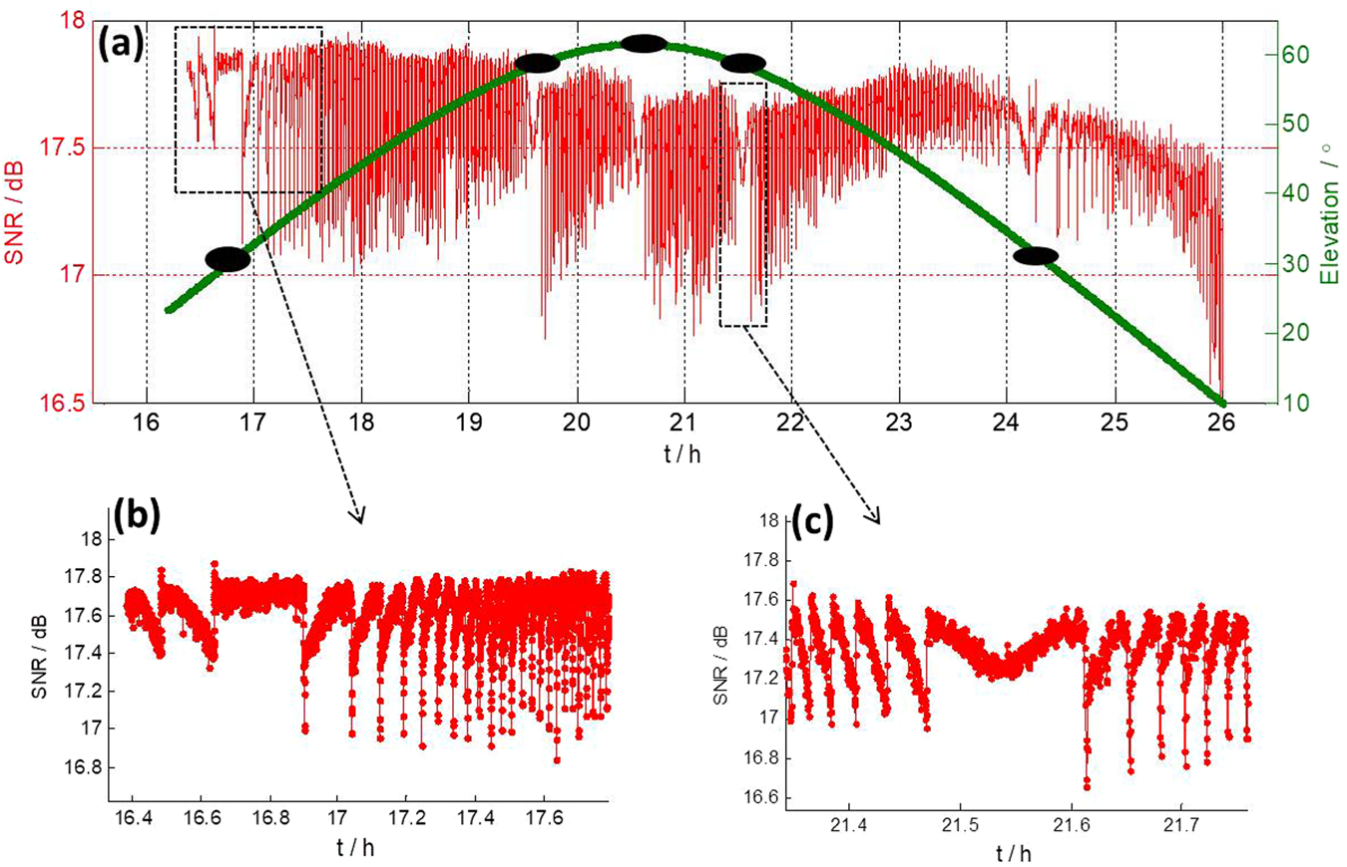
SNR of TM in X band Data transmission signals from CE3 lander on December 23, 2013. (**a**) SNR and elevation angle. The special regions are marked by black filled ellipses at different positions along elevation curve indicated by green solid line. (**b,c**) Zoomed in values of SNR from 2 special regions.

After CE3 landed on the Moon on December 14, 2013, it separated into a lander and a rover. The lander transmitted data transmission signals in X band 10 hours per day^10^. In this section we investigate the SNR of the lander over its long time observation. Figure 4 shows the SNR results of TM on December 23, 2013. In Fig. 4a, we present the SNR and elevation angle of TM together, because they were closely related behaviors.

In Fig. 4a, we identify 5 special regions as a function of time where SNR variations show a distinctive pattern, which has a symmetric structure and long variation period. Figure 4b,c are zoomed in values from 2 special regions, whose variation patterns are similar to the one of TM in Fig. 1a. We use black filled ellipses to mark the 5 special regions on the corresponding elevation angles, and find they lie at almost symmetric locations along the elevation path. We did the same investigation on azimuth angle, and found locations of special regions were not at all symmetric, which indicated SNR variations were possibly related only to elevation angle.

**Figure 5:**
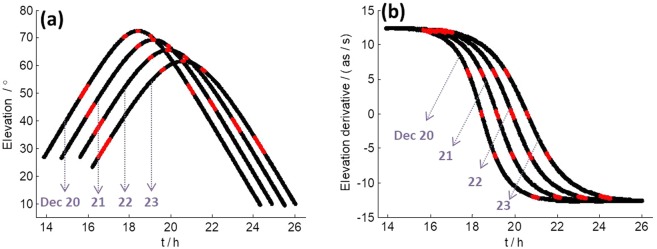
(**a**) Elevation angles of TM station from December 20 to 23, 2013. (**b**) Derivatives of the elevation angles. Areas in red dot correspond to special regions of SNR variation.

In order to know more about a possible relation between elevation and SNR variations, we compared elevation angles of 4 consecutive days (Fig. 5a). In Fig. 5a, elevation angles in red dots correspond to special regions of SNR variation. As we can see, they all correspond to symmetric locations along the elevation path. However, the elevation angles of such regions change day by day following a clear scheme. For example, the first special region declines around 7 degrees per day in elevation angle.

We then calculated the derivative of the elevation angle, which represents the elevation changing rate. Results are shown in Fig. 5b, which clearly shows all special regions are around the level of ± 12 as/s, ± 6 as/s, and 0 as/s, respectively. What’s more, we found that the derivatives of elevation angle of TM at 7.5 h on December 8, 2013 (Fig. 1a), and 8.5 h on October 26, 2014 (Fig. 2b) are both around 6 as/s. These findings show all of those SNR variations were caused by the same reason and it was closely related to elevation changing rate.

**Figure 6:**
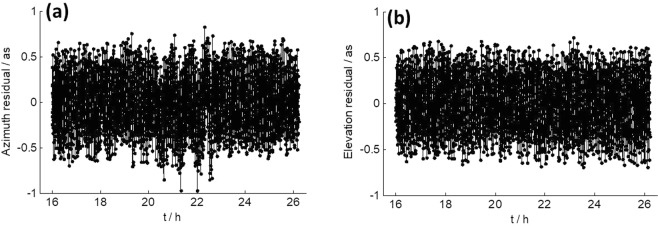
(**a**) Azimuth residuals and (**b**) elevation residuals after polynomial fitting of the pointing commands of TM when observing CE3 on December 23, 2013.

For the observations, the orbit of the spacecraft was predicted in advance, and theoretical elevation and azimuth angles for observing the spacecraft from TM station were calculated. Errors at level of a few tens of arcseconds in the theoretical elevation and azimuth angle (half power beam width of TM in X band: 120 as) may cause significant changes in the receiving power of TM and possibly result in the SNR variations shown in Fig. 4. To clarify this point, we obtained residuals of the theoretical elevation and azimuth angle after polynomial fitting. Figure 6 shows the results of observing CE3 from TM on December 23, 2013. As we can see, the residuals are within ±1 as, which is too small to cause large variation in SNR. We also obtained the residuals of observing CE3 from TM on December 8 and 9, 2013, and found they were all at the same level as in Fig. 6. Therefore, errors in the theoretical elevation and azimuth angles are excluded to be the reason.

## Pointing Error of TM

On April 29, 2015, TM performed observations on CE3, CE5T1, and quasars alternately before 16:00. After 16:00, TM pointed to the middle of CE3 and CE5T1, in order to receive signals simultaneously from both of them. On that day, CE3 transmitted X band DOR signals, and CE5T1 transmitted both X and S band signals.

**Figure 7:**
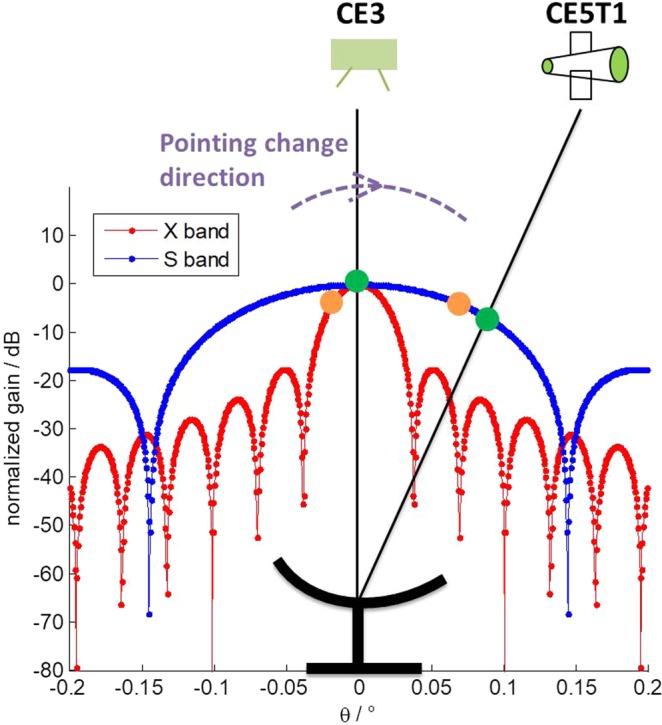
Sketch of TM pointing to CE3 and received S band signals from CE5T1 on April 29, 2015.

**Figure 8:**
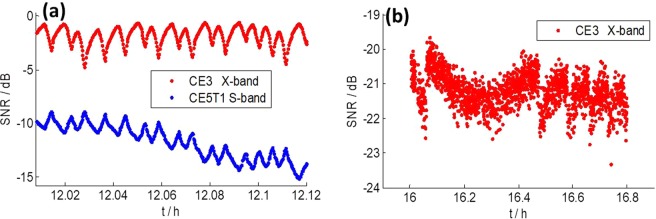
SNR of CE3 and CE5T1 on April 29, 2015. (**a**) TM pointed toward the CE3. (**b**) TM pointed toward the middle of CE3 and CE5T1.

The angular separation between CE5T1 and CE3 was very small (<0.15°) around 12:00, which resulted in S band signals from CE5T1 also being received while TM was pointing toward CE3. Figure 7 shows the telescope and target geometrical configuration and the theoretical gain curves in the direction of CE3 and CE5T1. As we can see, the S band beam is much wider than the X band one, allowing S band signals from CE5T1 to be received while TM was pointing toward CE3. In this case, we calculated the SNRs of CE3 and CE5T1 and show them together in Fig. 8a, in which red dots are the SNR of CE3 in X band signals and blue dots are the SNR of CE5T1 in S band signals. Surprisingly, the SNR of CE5T1 for S band signals also shows variation, which seems contradictory to what has been presented in Fig. 2a. What’s more, the variations of SNR for X band and S band signals show the same variation periods, but with exactly opposite variations. We can’t conceive of any other possibilities except for the actual pointing of TM went through major instabilities.

If actual pointing of TM suddenly turns a bit to the right side in Fig. 7, the antenna gains for receiving CE3 X band signals and CE5T1 S band signals would change from the values at green circles to the values at orange circles. As we can see, the antenna gain for receiving CE3 X band signals would decrease, whereas antenna gain for receiving CE5T1 S band signals would increase. Moreover, they change at the same time. This is in agreement with the variation shown in Fig. 8a.

As for why the variation in SNR of TM was not noticed in the S band (see Fig. 2a), it was possibly because TM was pointing precisely to CE5T1 during the observation, and the center of the S band beam is much larger and characterized by a much flatter gain over several tens of arcseconds comparing with X band. In this case, even though the actual pointing of TM went through instabilities, the antenna gain would only change by a small value and resulted in unnoticeable variation in SNR.

Assembling the information above, a pointing error of TM seems the most realistic reason, and it should be related to the elevation angle of TM, considering the analytic results in section 3. Since errors in the theoretical elevation and azimuth angles have been excluded to be the cause, it is reasonable to assume the pointing error originated from its control system or instrumental design. However, questions still remain as to why there was no such variation in SNR while TM was observing astronomical targets and why the variation disappeared in the years 2017 and 2018 (Fig. 3).

## Identification of the Error

### Log file of TM

The TM radio telescope generates a log file which records the actual pointing of TM when it performs observations, including the actual elevation and azimuth angles. They would be slightly different from the theoretical pointing values for the error of instrument. However, log files were not meant to be archived permanently, and very few log files taken in the years 2013, 2014 and 2015 are still available now. Fortunately, part of the data on April 29, 2015 was stored in a safe place, and investigations could be carried out.

As mentioned in section 4, TM pointed to the middle of CE3 and CE5T1 after 16:00 on April 29, 2015. Figure 8b shows the SNR of CE3 of X band DOR signals after 16:00. The SNR in Fig. 8b is low, but it clearly shows the same kind of variation as in Fig. 4b,c.

**Figure 9:**
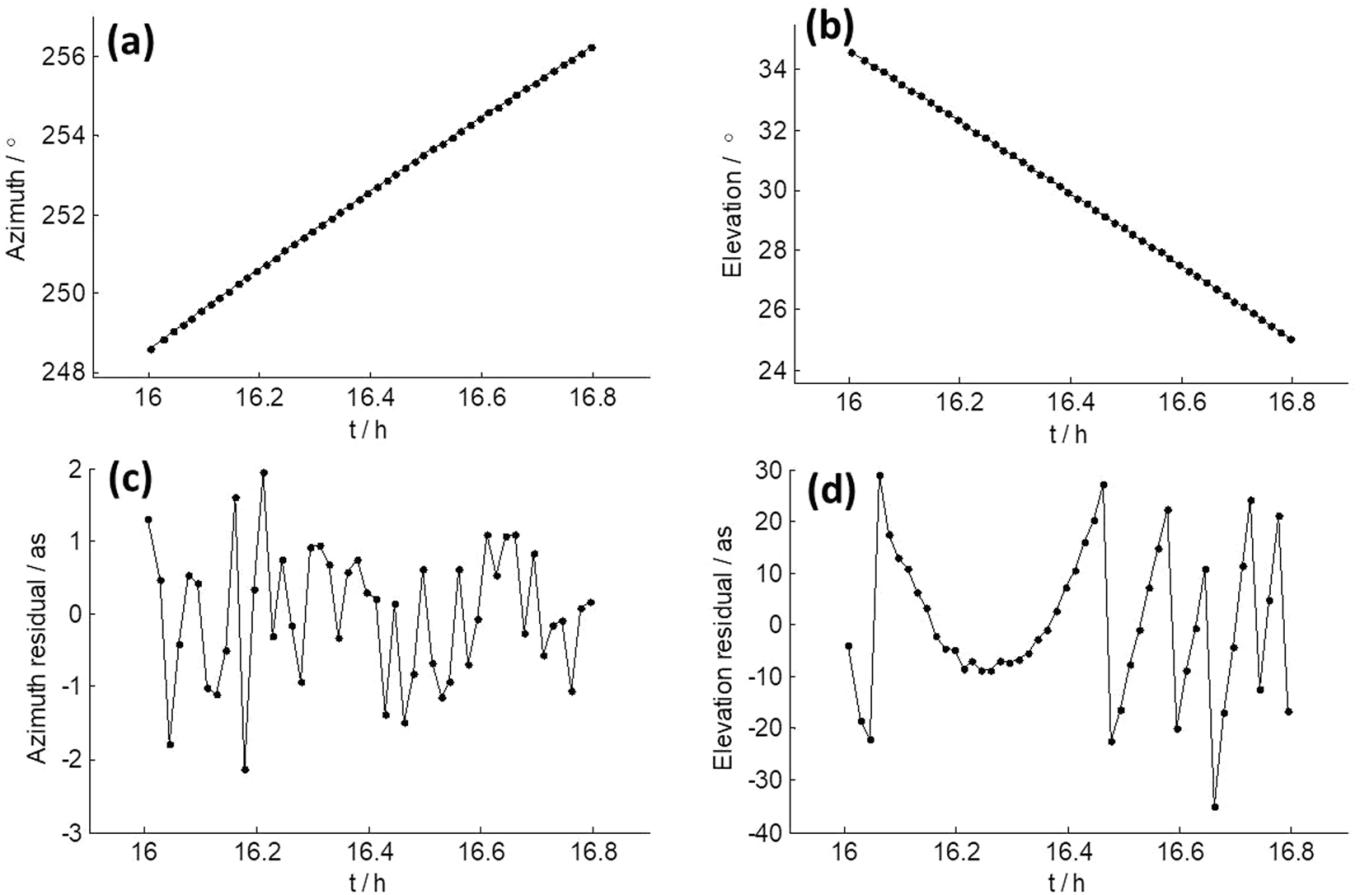
(**a,b**) Actual azimuth and elevation angle of TM on April 29, 2015 when observing the middle of CE3 and CE5T1. (**c,d**) Polynomial fitting residuals of azimuth and elevation.

We extracted actual azimuth and elevation angles from the log file of TM after 16:00, and show the results in Fig. 9a,b, respectively. Figure 9c,d are azimuth and elevation residuals after polynomial fitting of Fig. 9a,b. It is obvious that the elevation residuals in Fig. 9d change with the same pattern as the SNR in Fig. 8b, and show large variation in amplitude (±30 as). This could definitely cause the receiving power of TM to change significantly and therefore cause distinctive variation in SNR, as the half power beam width of TM in X band only extends to 120 as. On the other hand, azimuth residuals in Fig. 9c remain at a very low level (within a few arcseconds) and show no such variation at all. This is the very evidence that the SNR variation was caused by error in the actual elevation angle.

**Figure 10:**
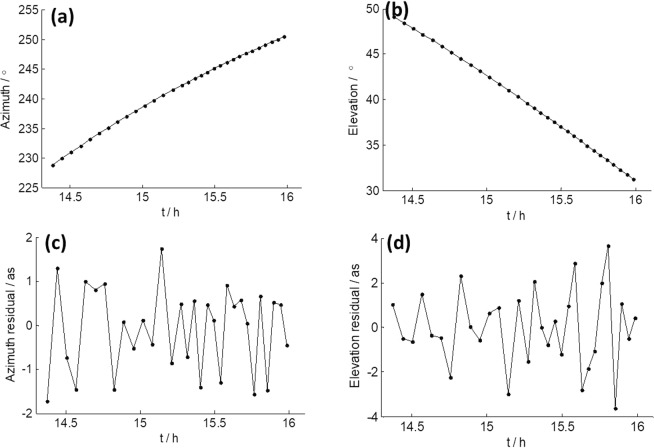
(**a,b**) Actual azimuth and elevation angle of TM on April 29, 2015 when observing quasar 1055 + 018. (**c,d**) Polynomial fitting residuals of azimuth and elevation.

We also extracted the actual azimuth and elevation angles from the log file when TM was observing quasar 1055018 before 16:00 of April 29, 2015. Results are shown in Fig. 10a,b, respectively. Figure 10c,d are azimuth and elevation residuals after polynomial fitting. As we can see, the azimuth and elevation residuals are both within a few arcseconds, whose influence on the receiving power of TM is so small that can be neglected. This indicates actual elevation angle did not have large errors when observing the quasar, which is in agreement with the results in Fig. 1c.

### Most likely reason—bug in elevation control software

It is now clear that the SNR variation was caused by the error in actual elevation angle, but it is still unknown why it would occur, and only occurred when observing the spacecraft. We investigated the software control system.

When tracking astronomical targets, the pointing of TM changes mainly because of the Earth’s rotation. However, when tracking a spacecraft, the pointing of TM changes not only because of the Earth’s rotation, but also because of the changing position of the spacecraft. For this reason, two sets of software were installed at the TM station, one for observing spacecrafts, the other for observing astronomical targets. We also notice that there was an update of the antenna control software in July, 2016.

Based on the above information, the most likely cause for the pointing error of TM is that there was a bug in the elevation control software which was used for observing spacecrafts, and it was fixed in the software update in July, 2016. Only this can explain why it only occurred when observing spacecrafts prior to 2016 (Fig. 1c), and disappeared in the years 2017 and 2018 (Fig. 3).

**Figure 11:**
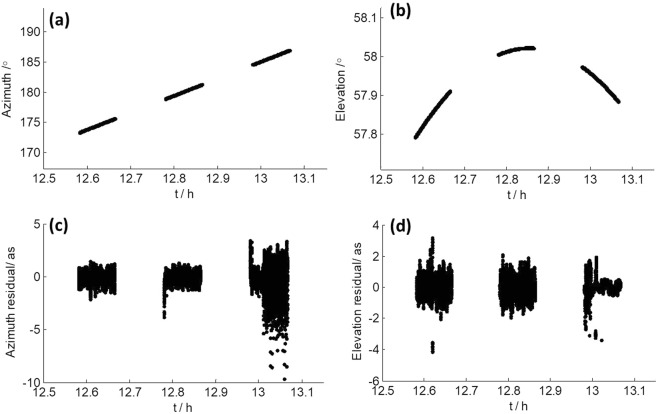
(**a,b**) Actual azimuth and elevation angle of TM on November 19, 2018 when observing CE3. (**c,d**) Polynomial fitting residuals of azimuth and elevation.

To demonstrate that the bug has been fixed, we extracted actual azimuth and elevation angles from the log file when TM was observing CE3 on November 19, 2018, and show them in Fig. 11a,b, respectively. The time range in Fig. 11 matches the time range in Fig. 3b. Figure 11c,d show the azimuth and elevation residuals after polynomial fitting, which are both within a few arcseconds. What’s more, the variation shown in Fig. 9d doesn’t show up in Fig. 11d anymore.

The software was rewritten in the update. Therefore it was not possible to pinpoint the bug. However, from the phenomenon shown in section 3, it looks like some sort of corrections in the elevation position were done every time the elevation changing rate increases/decreases by 6 as/s.

## Discussion

SNR variation would affect correlation phase accuracy. Considering that the SNR of carrier frequency is as high as 20 dB^11^, when the SNR drops by 4 dB because of pointing errors, the random error of the DOR group delay becomes 0.08 ns larger. However, the SNR drops less than 4 dB for most of the time. So overall, we estimate that the pointing error causes a few meters error on CE3 orbit determination. On the other hand, the SNR variation would affect the accuracy of Doppler frequency as well. We would suggest those groups who used the data from TM station observing spacecrafts before July, 2016 to double check their data in order to avoid misunderstanding.

To better track down and resolve similar issues in the future, we would give the following suggestions to the observational stations:

- Permanently archive at least one day log file per month.
- Archive all input control files, including spacecraft orbits used to generate pointing commands.
- Archive all versions of control software.

## Conclusion

The SNR of TM experienced peculiar variation in the years 2013, 2014, and 2015 when TM was observing spacecraft. The variation didn’t occur when TM was observing astronomical targets. Investigations discovered that the peculiar SNR variation was caused by pointing errors of TM, and indicated it originated from a bug in the elevation control software for observing spacecraft. The bug has been fixed in the software update in July, 2016, and the peculiar SNR variation disappeared in the years 2017 and 2018.
